# Monitoring clone dynamics and reversibility in clonal haematopoiesis and myelodysplastic neoplasm associated with PARP inhibitor therapy—a role for early monitoring and intervention

**DOI:** 10.1038/s41375-023-02040-6

**Published:** 2023-11-17

**Authors:** Ellen Nuttall Musson, Rowan E. Miller, Marc R. Mansour, Michelle Lockley, Jonathan A. Ledermann, Elspeth M. Payne

**Affiliations:** 1 UCL Cancer Institute, London, UK; 2 University College London Hospitals NHS Foundation Trust, London, UK; 3 Centre for Cancer Genomics and Computational Biology, Barts Cancer Institute, Queen Mary University of London, London, UK

**Keywords:** Adverse effects, Myelodysplastic syndrome

## To the Editor:

Poly ADP-ribose polymerase inhibitors (PARPi) are widely used as maintenance treatment for ovarian cancer (OC) after response to platinum chemotherapy in the first-line setting and recurrent disease. Their use has significantly improved outcomes, particularly in patients with BRCA mutations [1] in whom first-line PARPi maintenance therapy increases progression-free survival from 13.8 to 56.0 months (HR:0.33; 95% CI:0.25–0.43). However, a recent meta-analysis showed that PARPi-therapy significantly increases the risk of therapy related myeloid neoplasms (tMN), myelodysplastic neoplasm (MDS) and acute myeloid leukaemia with poor outcomes and these findings have been expanded using pharmacovigilance data [2, 3]. Cytopenias occur frequently on PARPi-therapy (with grade 3–4 cytopenias reported in up to 34% [4]), complicating identification of more serious haematological toxicity (HT).

We present a case series of OC patients receiving PARPi-therapy referred for haematological review for beyond-expected HT. We show using sequential molecular and haematological monitoring before and after PARPi cessation that morphological, Full Blood Count (FBC) and clonal abnormalities can be reversed on PARPi discontinuation, potentially mitigating the risk of life-threatening tMNs in some patients.

Six patients with stage III/IV high-grade OC were referred to haematology between February 2017 and August 2021 (mean age (SD): 67.3 (7.4) years, median PARPi duration: 18 months (range: 4–86)). Reasons for referral are detailed in Table 1. All patients had received prior platinum-based chemotherapy and were screened with myeloid Next Generation Sequencing (m-NGS) in peripheral blood (PB) or bone marrow aspirate (BMA) at initial assessment. All samples were analysed in the same specialist integrated haematological malignancy diagnostic laboratory utilising World Health Organisation 2022 diagnostic criteria. All BMAs/m-NGS analyses were performed at the discretion of the treating clinicians. Adverse events are reported according to Common Terminology Criteria for Adverse Events criteria. Informed consent was obtained from all patients.

**Table 1:** Clinical details and laboratory parameters.

Patient number	Germline BRCA genotype	Number of prior lines of platinum chemotherapy	PARPi	PARPi duration at referral (months)	Age at haematological referral (years)	Reason for haematology referral	Full Blood Count at haematological referral	Diagnostic Myeloid NGS Panel Mutations (VAF)	Diagnosis (2022 WHO Criteria)	Revised International Prognostic Scoring System	Full Blood Count at last follow up	
											Full Blood Count results	Months post PARPi cessation
1	BRCA1 mutation	2	Rucaparib	15	75	Transfusion dependence, progressive thrombocytopenia	Hb: 117 WCC: 4.82 Plt:45 MCV: 99.2 RDW: 15.1	PPM1D c.1281G>A, p.Trp427Ter (15%), TP53 copy number loss detected.	MDS-LB	4	Hb: 122 WCC: 7.36 Plt:170 MCV: 92.8 RDW: 12.9	75
2	Wildtype	2	Olaparib	18	67	Macrocytosis, intermittent cytopenias	Hb: 99 WCC: 3.44 Plt: 182 MCV: 119.5 RDW: 13.2	DNMT3A c.2095G>T, p.(Gly699Cys) (16%)	MDS-LB	2	Hb: 117 WCC: 5.51 Plt: 238 MCV: 91 RDW: 15.3	32
3	BRCA1 mutation	1	Olaparib	86	72	Persistent unexplained macrocytosis	Hb: 118 WCC: 6.85 Plt: 189 MCV: 108.1 RDW: 14.3	TP53 c.701A>G, p.(Tyr234Cys) (10.5%), DNMT3A c.2204A>G, p.Tyr735Cys (14%).^a,b^	CCUS	N/A	Hb: 143 WCC: 6.79 Plt: 326 MCV: 92 RDW: 12.4	23
4	Wildtype	2	Niraparib	4	73	Prolonged thrombocytopenia progressing to macrocytic pancytopenia	Hb: 95 WCC: 2.14 Plt: 48 MCV: 109.9 RDW: 15.7	SMC3A c.1964–1_1977del (splice-acceptor site) (20%)^a,b^	CCUS	N/A	Hb: 114 WCC: 6.31 Plt: 217 MCV: 106.6 RDW: 12.2	14^c^
5	BRCA2 mutation	2	Niraparib	24	60	Macrocytosis, recurrent nucleated red blood cells on blood film	Hb: 142 WCC: 5.96 Plt: 342 MCV: 99.8 RDW: 13.8	PPM1D c.1384C>T, p.Gln462Ter (3%)	CHIP	N/A	Hb: 143 WCC: 6.46 Plt: 371 MCV: 93.6 RDW 13.9	N/A
6	BRCA2 mutation	1	Olaparib	18	57	Persistent anaemia with transfusion requirement	Hb: 72 WCC: 6.46 Plt: 265 MCV: 103 RDW: 20.8	No pathogenic mutations^a^	N/A	N/A	Hb: 89 WCC: 9.05 Plt: 292 MCV: 118.9 RDW: 18.8	N/A

*Hb* haemoglobin (g/L), *WCC* white cell count (×10^9^/L), *Plt* platelet count (×10^9^/L), *MCV* mean cell volume (fL), *RDW* red cell distribution width (%).

^a^PB.

^b^Variant of uncertain significance.

^c^In context of progressive ovarian cancer.

**Fig. 1:**
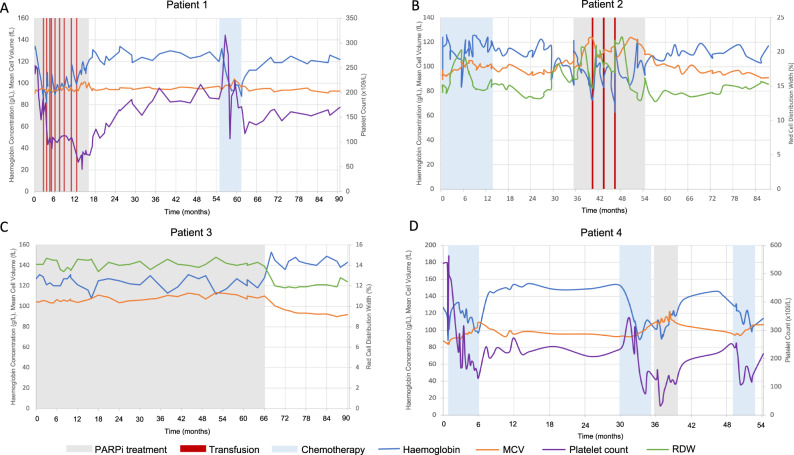
Individual laboratory parameters over time in cytopenic patients in relation to PARPi treatment. **A** Patient 1: haemoglobin concentration, mean cell volume (MCV), platelet count and transfusion requirement in relation to chemotherapy (blue) and PARPi treatment (grey). **B** Patient 2: haemoglobin concentration, MCV, red cell distribution width (RDW) and transfusion requirement in relation to chemotherapy and PARPi treatment. **C** Patient 3: haemoglobin concentration, MCV and RDW in relation to PARPi treatment. **D** Patient 4: haemoglobin concentration, MCV and platelet count in relation to chemotherapy and PARPi treatment.

Five patients had clonal pathology (CP), four of whom had single-nucleotide variants typical of platinum-exposure defined by the involved gene [5] or platinum-associated mutational signatures [6]. Clinical details including BRCA-status, haematological diagnosis and m-NGS results are detailed in Table 1. PARPi-therapy was discontinued in four patients with CP, with resolution or improvement of FBC and m-NGS abnormalities (variant allele frequencies (VAFs) and/or copy number variations (CNVs)) in three patients. Detailed descriptions of the cases with CP are provided below. Figure 1 shows longitudinal laboratory parameters in relation to PARPi-therapy for patients with clonal cytopenias.

*Patient 1* was diagnosed with stage IIIb serous ovarian adenocarcinoma, treated with surgery and adjuvant carboplatin and paclitaxel. She underwent resection of recurrent disease 3 years later followed by single-agent carboplatin. Six years after initial diagnosis she had disease progression. She was commenced on CLOVIS Oncology Phase I trial with rucaparib for 19 cycles but developed grade 2–3 thrombocytopenia and grade 3 anaemia, requiring 14 red cell transfusions in 10 months. Rucaparib was discontinued and she was referred to haematology. A BMA showed myeloid and megakaryocytic dysplasia (blast count: 2%). Chromosomal microarray analysis showed cryptic deletions at 16p11.2 (including MAPK3), loss of the TP53 region at 17p13 and deletion at 20q11.22 region. M-NGS detected a PPM1D variant (VAF 15%) and a TP53 copy number loss. She was diagnosed with MDS with low blasts (MDS-LB). Rucaparib was discontinued and she became transfusion independent (Fig. 1A). A repeat BMA 6 months later showed no dysplasia and the TP53 deletion was no longer detected (confirmed by fluorescence in situ hybridisation on 100 cells). Four years after initial BMA, PB m-NGS showed a persistent PPM1D mutation (VAF 9%) with no CNVs detected.

*Patient 2* was diagnosed with stage IIIb serous OC, treated with surgery and carboplatin, paclitaxel and bevacizumab (BCP, ICON8-B trial). She relapsed 14 months later and was treated with carboplatin and liposomal-doxorubicin followed by maintenance olaparib (ICON9 trial). Olaparib was dose-reduced after 10 months due to grade 3 anaemia with a transfusion requirement (5 units in 6 months), with improvement in haemoglobin concentration. A marked macrocytosis and elevated red cell distribution width (RDW) persisted, prompting referral to haematology after 18 months of PARPi-therapy. BMA showed moderate erythrodysplasia with no blast excess consistent with MDS-LB. M-NGS detected a DNMT3A mutation (VAF 16%). Olaparib was discontinued resulting in transfusion independence and normalisation of mean cell volume (MCV) and RDW. A repeat BMA 6 months later showed multilineage dysplasia and a modest reduction in the detectable DNMT3A variant (VAF 10%).

*Patient 3* was diagnosed with stage IV serous ovarian adenocarcinoma. She received six cycles of carboplatin and paclitaxel followed by olaparib maintenance in the SOLO-1 trial. She had unexplained macrocytic anaemia and was referred to haematology 86 months after commencing PARPi-therapy. PB m-NGS showed mutations in TP53 (VAF 10.5%) and DNMT3A (VAF 14%). She was diagnosed with clonal cytopenia of uncertain significance (CCUS) and olaparib was discontinued. Her haematological parameters improved (Fig. 1C). On repeat PB m-NGS 20 months after olaparib cessation the TP53 variant was detectable at a markedly reduced VAF (<1%). The DNMT3A variant remained present at a stable VAF (16%).

*Patient 4* was diagnosed with stage IIIC serous OC and was treated with surgery and adjuvant BCP. She had progressive disease 2 years after completing first-line chemotherapy and was treated with 5 cycles of carboplatin and liposomal-doxorubicin with partial response (PR). She had grade 1 thrombocytopenia following this. She commenced niraparib which was stopped after 3 weeks due to grade 3 thrombocytopenia. Her counts recovered and she restarted niraparib at a reduced dose 6 weeks later. She had persistent grade 1 thrombocytopenia progressing to mild macrocytic pancytopenia and was referred to haematology. PB m-NGS detected a variant in SMC3A at a splice-acceptor site (VAF 20%). She was diagnosed with CCUS. Niraparib was discontinued and her FBC normalised. One year later, PB m-NGS showed a stable SMC3A variant and a new PPM1D variant (p.Lys535Ter (VAF 18%)) and she was diagnosed with progressive OC and received cisplatin-therapy.

*Patient 5* was diagnosed with stage IV serous papillary OC, treated with single-agent carboplatin and interval debulking surgery, completing 6 cycles of carboplatin postoperatively. She had disease recurrence 5 years later and she was treated with 6 cycles of carboplatin and liposomal-doxorubicin with good PR and received niraparib for 2 years. She had no cytopenias but had numerous circulating nucleated red blood cells on blood film microscopy, prompting referral to haematology. BMA showed no dysplasia or fibrosis and normal cytogenetic investigations. M-NGS detected a PPM1D variant (VAF 3%). She was diagnosed with Clonal Haematopoiesis of Indeterminate Potential (CHIP). Given her morphologically normal BMA and normal haematological parameters, a multidisciplinary team (MDT) decision was made to continue PARPi-treatment with regular FBC and m-NGS monitoring. Repeat PB m-NGS 6 months later showed a stable PPM1D variant (VAF 3%) with no new variants.

Outcomes following haematological treatment for PARPi-associated t-MN are dismal [7]. We highlight the importance of monitoring patients with disproportionate HT on PARPi-therapy, as a predictive indicator for clonal pathology. Crucially, using serial monitoring this series demonstrates potential for reversibility in haematological, molecular and morphological abnormalities in PARPi-associated HT, even in those with a formal diagnosis of MDS.

The risk of myeloid neoplasm in patients with OC is multifaceted. Platinum-based therapy is associated with a significantly increased risk of clonal haematopoiesis with malignant driver mutations [5] and there is a dose-dependent relationship between the risk of tMNs and cumulative platinum exposure in patients with OC [8]. BRCA mutation status is also relevant; germline BRCA variants are enriched in primary MDS/AML indicating that they may represent a germline predisposition [9]. CHIP is more prevalent in patients on PARPi-therapy compared to other oncological treatments [10], suggesting that PARPi-therapy confers a selective advantage for CHIP clones following chemotherapy. With improved survival post platinum exposure and the use of PARPi first-line in OC, these risks require important consideration.

We demonstrate the variability in the nature and degree of haematological abnormalities observed in PARPi-associated CP. Macrocytosis is a common feature in PARPi-associated anaemia [11] and we have observed changes in RDW (Fig. 1) which correlate with PARPi exposure. Modelling studies have identified red cell indices as predictive of a diagnosis of MDS [12] and progression from CHIP to myeloid neoplasm [13]. Population wide analysis of the specific features of PARPi associated HT may help to identify those that indicate clonal pathology and warrant further research.

The role of m-NGS is recognised in the diagnosis and prognostication of MDS [14]. We demonstrate the utility of molecular monitoring for CP evolution during PARPi-therapy. Pre-existing TP53 CH variants are associated with t-MNs after rucaparib treatment [15], also highlighting the potential role for NGS analysis prior to PARPi commencement. This may also identify pre-existing CHIP unrelated to chemotherapy exposure, which could be the case for the DNMT3A variant identified in Patient 3. Prospective studies are needed to systematically assess the effects of PARPi on pre-existing and chemotherapy-associated CHIP, CCUS and the evolution of MDS to stratify those at greatest risk of tMN and inform oncological treatment decisions. Importantly, the risks and benefits of stopping PARPi versus risk of tMN need to be carefully assessed on a case-by-case basis for which development of an MDT pathway may be helpful.

## Data Availability

Data available on reasonable request from the authors.
